# Estimating the Size of the Aedes Mosquitoes’ Population Involved in Outbreaks of Dengue and Chikungunya Using a Mathematical Model

**DOI:** 10.1007/s11538-025-01489-z

**Published:** 2025-08-08

**Authors:** Francisco Antônio Bezerra Coutinho, Marcos Amaku, Fernanda Castro Boulos, José Alfredo de Sousa Moreira, Eliana Nogueira Castro de Barros, Esper Georges Kallas, Eduardo Massad

**Affiliations:** 1 School of Medicine, University of São Paulo, São Paulo, Brazil; 2 Instituto Butantã, São Paulo, Brazil; 3 Fundação Getúlio Vargas, São Paulo, Brazil; 4 School of Public Health, University of São Paulo, São Paulo, Brazil

**Keywords:** Aedes aegypti, Dengue, Chikungunya, Vector-borne infections, Mathematical modeling, Epidemiology

## Abstract

*Aedes aegypti* continues to cause many cases of dengue, chikungunya and Zika fever in affected areas of the tropical world. After being eradicated from Brazil in the decades of 1940 and 1950, *Aedes aegypti* returned with full force in the early 1970s. Knowing the total number of mosquitoes transmitting Aedes-borne infections is crucial for quantifying the intensity of transmission of these infections. In this paper, we propose a model to estimate the distribution of the number of Aedes mosquitoes’ populations during an outbreak of either dengue or chikungunya. The model assumes that the mosquitoes’ distribution follows a Gaussian Mesa Function (GMF), which has 5 parameters and allows for variable asymmetry. These 5 parameters are adjusted so that they fit indirectly, from a modified Ross‒Macdonald model, the incidence of dengue or chikungunya infections (see main text). Therefore, the observed incidence becomes a function of the parameters of the GMF. We illustrate the model with dengue and chikungunya data from 5 cities in the state of Minas Gerais in the southeastern region of Brazil for the 2023–2024 transmission season. The model shows that it is possible to estimate the size of the mosquitoes’ population from incidence data, circumventing the logistic hurdles involved in the actual counting of mosquitoes. This is the most important practical contribution of this paper. The paper also contains several theoretical innovations, such as a modification of the Ross‒Macdonald model, which is usually presented for a constant mosquitoes’ population, which, of course, is very unrealistic.

## Introduction

In the decades of 1940 and 1950, Brazil adopted strict measures to control *Aedes aegypti*, which, at the time, transmitted mainly urban yellow fever. In 1958, the World Health Organization (WHO) declared Brazil mosquito-free (Kotsakiozi et al. 2017; Fiocrus 2024). However, *Aedes aegypti* has continued to exist in other neighboring countries and even in small quantities in Brazil. It probably returned in full force to Brazil in the early 1970s (Kotsakiozi et al. 2017) because of population flow on the continent and the relaxation of control measures against yellow fever. Today, the mosquito is present in all Brazilian states (MOH Brazil 2024a).

*Aedes aegipti*, in 2024 alone, caused 6,396,442 dengue cases (MOH Brazil 2024b), 380,740 chikungunya cases (MOH Brazil 2024c), and 34,006 Zika fever cases (MOH Brazil 2024d). The total number of cases of dengue, chikungunya and zika in the last decade has reached approximately 25 million in Brazil [the total number of deaths due to Aedes virus-transmitted infections in recent decades has reached approximately 10 thousand people (BBC News Brazil 2024)].

Several factors have made the control of *Aedes aegiptii* very difficult, including its highly anthropophilic behavior, ability to lay desiccation-resistant eggs, high passive dispersal (by winds, for example) and tendency to rapidly evolve to pesticide resistance (Bass and Field 2011; Martins et al. 2009).

One of the key factors determining the intensity of transmission of Aedes-borne infections is the size of the mosquitoes’ population (Massad et al. 2017). This variable, however, is difficult to estimate and, therefore, poorly studied (Massad et al. 2017). In fact, the incidence of Aedes-transmitted infections show a marked seasonality, driven by the oscillation in mosquitoes number, which, in turn, is due to climatic factors.

In a previous paper, Massad et al. (2017) calculated the size of Aedes mosquitoes’ populations on the basis of dengue incidence data during an outbreak. The authors’ method consisted of fitting a continuous curve to the incidence data and assuming that the outbreak was of the form of a classical Ross‒Macdonald model (Amaku et al. 2016). After a series of mathematical manipulations, the authors estimated the number of susceptible, latent and infected mosquitoes. This method, however, is dependent on curve fitting data of incidence, because the method is dependent on derivatives of it, and furthermore uses a simple Ross‒Macdonald model that is not completely adequate for outbreaks.

In this paper, we present an alternative method for estimating the size of Aedes mosquitoes’ populations during an outbreak of either dengue or chikungunya fever. In Sect. 2 we describe this method. In Sect. 3, we present the results of the model for five cities with high incidences of dengue and chikungunya from a state in the southeastern region of Brazil. In Sect. 4, we discuss our findings and present a general conclusion.

## Methods

**Fig. 1:**
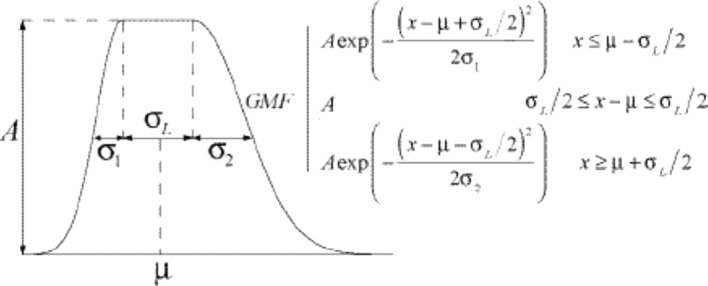
Gaussian Mesa function distribution of mosquitoes. From Dubois et al. (2007). In the figure, the abscissa represents time and ordinate Numbers (Color figure online)

The mosquitoes’ population is divided in susceptible ${S}_{m}\left(t\right),$ latent ${L}_{m}\left(t\right),$ and infected ${I}_{m}\left(t\right).$ The method assumes that the number of susceptible Aedes mosquitoes causing an outbreak of dengue or chikungunya is distributed in time as a Gaussian Mesa function (Dubois et al. 2007), which has the form shown in Fig. 1 and corresponds to Eq. (1) and which represents the seasonal fluctuation of mosquitoes’ population.

As mentioned above, we assume that the GMF is the number of susceptible mosquitoes, ${S}_{M}\left(t\right)$, (prevalence) and it enters into a modified Ross‒Macdonald model (Amaku et al. 2016), described in Eqs. (2–12). In the GMF expression, parameter $A$ represents the amplitude of the mosquitoes’ prevalence curve; parameters ${\sigma }_{i} (i=L, 1, 2)$ represent the variability in the mosquitoes’ numbers around the mean, represented by parameter $\mu$. Since ${S}_{M}\left(t\right)$ is rarely measured, it is important to see how the human incidence ($Incidence\left(t\right)$), see equations below) is influenced by the parameters of the GMF. Later in the paper, we show a sensitivity analysis of the total number of human cases with respect to each parameters of the GMF.

**Fig. 2:**
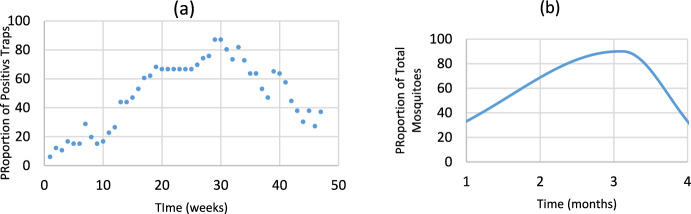
Mosquito distribution. In **a** the actual proportion of ovitraps in Belo Horizonte, Minas Gerais (Eiras, personal communication); **b** the GMF for the city of Barretos, São Paulo (Color figure online)

Figure 2 is intended only to show that the GMF function can be adapted to mimic incidence data in a qualitatively way.

The model is described by the following system of equations:

1 $${S}_{m}(t)=\left[\begin{array}{c} A*exp\left[-\frac{{\left(t-\mu +\frac{{\sigma }_{L}}{2}\right)}^{2}}{2{\sigma }_{1}}\right], for t\le \mu -\frac{{\sigma }_{L}}{2}\\ A, for \frac{{\sigma }_{L}}{2}\le t-\mu \le \frac{{\sigma }_{L}}{2}\\ A*exp\left[-\frac{{\left(t-\mu -\frac{{\sigma }_{L}}{2}\right)}^{2}}{2{\sigma }_{2}}\right], for t\ge \mu -\frac{{\sigma }_{L}}{2}\end{array}\right.$$

2 $$\frac{d}{dt}{L}_{m}\left(t\right)=ac\frac{{I}_{h}\left(t\right)}{{N}_{h}}{S}_{m}(t)-\left({\mu }_{m}+{\gamma }_{m}\right){L}_{m}(t)$$

3 $$\frac{d}{dt}{I}_{m}\left(t\right)={\gamma }_{m}{L}_{m}\left(t\right)-{\mu }_{m}{I}_{m}(t)$$

4 $$\frac{d}{dt}{S}_{h}\left(t\right)=-ab\left[1-\frac{H}{{N}_{H}}\right]{I}_{m}\left(t\right)\frac{{S}_{h}\left(t\right)}{{N}_{h}}+{\mu }_{h}\left({I}_{h}\left(t\right)+{R}_{h}(t)\right)$$

5 $$\frac{d}{dt}{I}_{h}\left(t\right)= ab{\left[1-\frac{H}{{N}_{H}}\right]I}_{m}\left(t\right)\frac{{S}_{h}\left(t\right)}{{N}_{h}}-\left({\mu }_{h}+{\gamma }_{h}\right){I}_{h}(t)$$

6 $$\frac{d}{dt}{R}_{h}\left(t\right)={\gamma }_{h}{I}_{h}\left(t\right)-{\mu }_{h}{R}_{h}(t)$$

7 $$Incidence\left(t\right)=ab{I}_{m}\left(t\right)\frac{{S}_{h}\left(t\right)}{{N}_{h}}$$

8 $${N}_{M}\left(t\right)={S}_{M}\left(t\right)+{L}_{M}\left(t\right)+{I}_{M}(t)$$

9 $${N}_{H}={S}_{H}\left(t\right)+{I}_{H}\left(t\right)+{R}_{H}(t)$$

10 $$Human Cases (t)=\underset{0}{\overset{t}{\int }}Incidence\left(s\right)ds$$

11 $$H=\sum_{{t}_{init}}^{t-1}Cases$$

The term $\left[1-\frac{H}{{N}_{H}}\right]$ takes into account the cumulative number of past cases in the whole history of the infections, from the reported cases to the Ministry of Health database, approximately mimicking herd immunity and, therefore, corrects the number of susceptible individuals in the population. As dengue is a more antique disease in Brazil than chikungunya, its time series is much longer. Moreover, dengue is more transmissible than chikungunya. Therefore, the term $\left[1-\frac{H}{{N}_{H}}\right]$ is significant for dengue but is negligible for chikungunya.We describe the model (2–12) variables and parameters in Table 1.

**Table 1:** Model’s variables and parameters

Variables	
Variable	Biological meaning
${S}_{M}(t)$	Susceptible mosquitoes (GMF)
${L}_{M}(t)$	Latent mosquitoes
${I}_{M}(t)$	Infected mosquitoes
${S}_{H}(t)$	Susceptible humans
${I}_{H}(t)$	Infected humans
$R(t)$	Recovered humans
${N}_{H}$	Total human population (assumed constant)
$H$^+^	Sum of cases from the moment the infection was introduced (${t}_{init}$) until $t-1$

Parameters*		
Parameter	Biological meaning	Values^a^
$$a$$	Mosquitoes’ biting rate	30 months^−1^
$$c$$	Mosquitoes’ susceptibility to the infections	0.6 for chikungunya
		0.7 for dengue
$${\mu }_{M}$$	Mosquitoes’ natural mortality rate	0.1 months^−1^
$${\gamma }_{M}$$	Rate of evolution from latent to infected mosquitoes^$^	2 months^−1^ for chikungunya
		1.5 months^−1^ for dengue
$$b$$	Humans’ susceptibility to the infections	0.6 for chikungunya
		0.7 for dengue
$${\mu }_{H}$$	Humans’ natural mortality rate	1.2 × 10^–3^ months^−1^
$${\gamma }_{H}$$	Humans’ recovery rate from the infections	2 months^−1^

^+^The number of cumulative cases assumes immunity for life and deaths are neglected

^*^The parameters meaning for GMF are shown in Fig. 1

^$^The inverse of the mosquitoes’ extrinsic incubation period

^a^Data from Massad et al. (2008) and references cited in it

The model is as follows:

- *Step 1* The outbreak incidence data of dengue or chikungunya (blue dots in the figures below) in each given city for a given outbreak season are obtained from the Brazilian Ministry of Health Database (SINAN MOH Brazil. 2024) in a monthly base;
- *Step 2* We use the history of the diseases obtained from the chosen city to calculate the values of ${S}_{H}\left(0\right)={N}_{H}\left[1-\frac{H}{{N}_{H}}\right]$ and ${R}_{H}(0)$;
- *Step 3* Then, we used a computer algorithm to find out the parameters of the GMF, fitted by least squares technique, so that with these parameters Model (2–12) produces the incidence of dengue or chikungunya that best fits the observed data. Therefore, the incidence becomes a function of the parameters of the GMF. In the Results section, we show how good is the fitting of the calculated incidence to the observed data, and also we carry out a sensitivity analysis of the number of cases, during the outbreak, for each GMF parameters;
- *Step 4* The model also gives the number of mosquitoes (susceptible, latent and infected) that are involved in the outbreak. We noted that the number of human susceptibles varies little during the outbreaks studied.

**Fig. 3:**
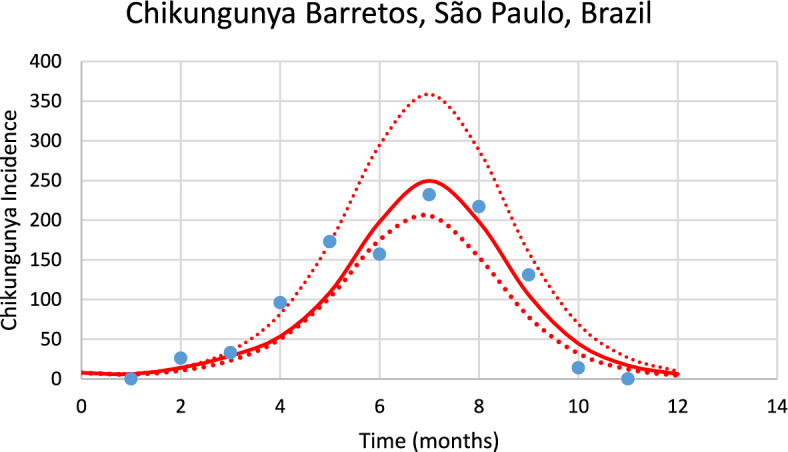
Model fitting performance for the chikungunya outbreak between September 2022 and August 2023 in the city of Barretos in the southeastern state of São Paulo, Brazil. Blue dots represent real data and red line the model’s result and dotted line the 95% Confidence interval [*Incidence* (*t*) as in Eq. (1)] (Color figure online)

Figure 3 shows an example of the model’s performance for the chikungunya outbreak in the period between September 2022 and August 2023 in the city of Barretos in the southeastern state of São Paulo, Brazil.

To illustrate the model, we analyzed outbreaks of dengue and chikungunya in five cities in the State of Minas Gerais, Southeast Brazil. We chose this state because it had the highest incidence of chikungunya in the country in 2024 and one of the highest incidences of dengue in the country in the same year. The chosen cities are Coronel Fabriciano (population 104,736), Teófilo Otoni (population 137,418), Paracatú (population 94,023), Pirapora (population 55,606), and Aimorés (population 25,269). The size of a city is important. The number of habitants cannot be greater than approximately 100,000 and not smaller than approximately 20,000 because we assume that the whole population of humans is involved in the outbreak. This is partially justified because we assume that the mosquitoes can reach any person because humans move around the city.

Then, we calculated the number of susceptible mosquitoes infected with both dengue and chikungunya viruses for each city and correlated them with the observed incidences of dengue and chikungunya, respectively. From these analyses, we found that each infected mosquito produces roughly the same number of cases for both infections. The objective of this analysis is to estimate the slope of the line correlating the number of infected mosquitoes, *I*_*M*_ (t), with the incidences of both dengue and chikungunya, denoted *Inc*_*dengue*_(*t*) and *Inc*_*chik*_(*t*), respectively. These variables are related to each other via the following equation:

12 $${Inc}_{i}=\left\{a{b}_{i}\left[1-\frac{H}{{N}_{H}}\right]\frac{{S}_{{H}_{i}}\left(t\right)}{{N}_{H}}\right\}{I}_{{M}_{i}}\left(t\right), i=dengue, chikungunya$$

Hence, the slope of the correlation lines corresponds to the first term of Eq. (12)

13 $$a{b}_{i}\left[1-\frac{H}{{N}_{H}}\right]\frac{{S}_{{H}_{i}}\left(t\right)}{{N}_{H}}\cong a{b}_{i}\left[1-\frac{H}{{N}_{H}}\right]\frac{{S}_{{H}_{i}}\left(0\right)}{{N}_{H}}$$

because ${S}_{{H}_{i}}\left(t\right)\cong {S}_{{H}_{i}}\left(0\right)$, that is, the observe variation in human susceptibles is very small (less than 1%).

Finally, we also compared the model’s estimation of the number of Aedes mosquitoes with that empirically measured in the city of Foz do Iguaçu, a city with 285,415 inhabitants, localized in the State of Paraná, South Region of Brazil (Rodrigues et al. 2017).

**Fig. 4:**
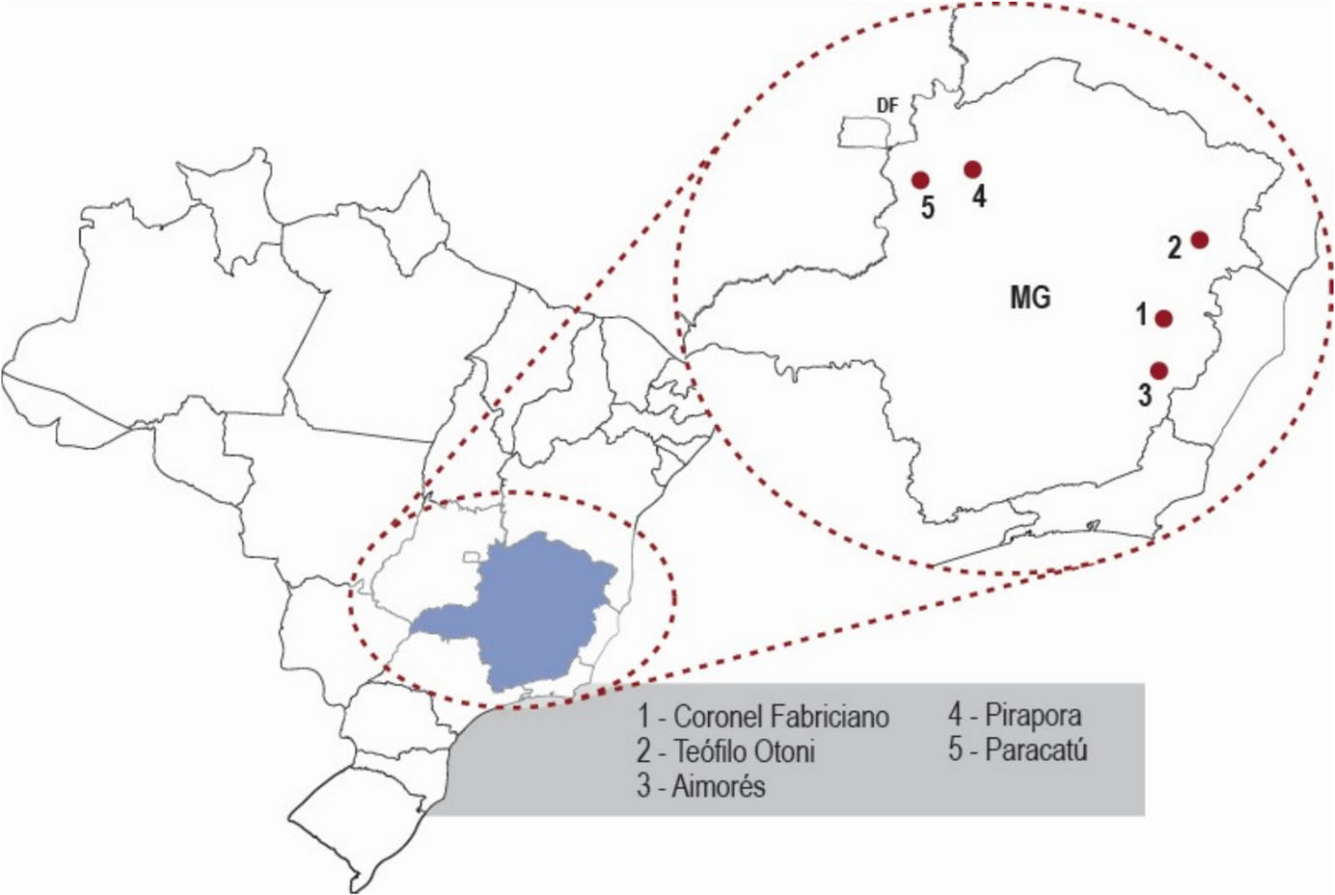
Approximate localization of the five cities studied on the map of Minas Gerais State, Brazil (Color figure online)

Figure 4 shows the approximate locations of the five cities on the map of Minas Gerais State.

## Results

In what follows, we describe our analyses for the five cities cited above (Fig. 4).

### Coronel Fabriciano City

**Fig. 5:**
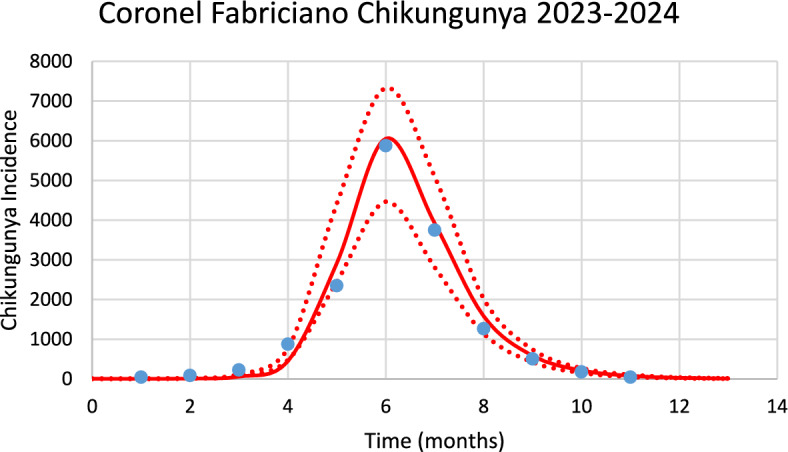
Model’s performance in fitting real data of chikungunya incidence for the city of Coronel Fabriciano in the 2023–2024 season of transmission (R^2^ = 0.92). Blue dots represent real data, red line the model’s result and dotted line the Confidence interval varying the size of the mosquitoes’ prevalence by 5% [*Incidence* (*t*) as in Eq. (1)] (Color figure online)

Figure 5 shows the model’s performance in fitting the real data of chikungunya incidence for Coronel Fabriciano in the 2023–2024 season of transmission.

The number of observed chikungunya cases during the outbreak was 14,050, whereas the total number of calculated cases was 13,561.

**Fig. 6:**
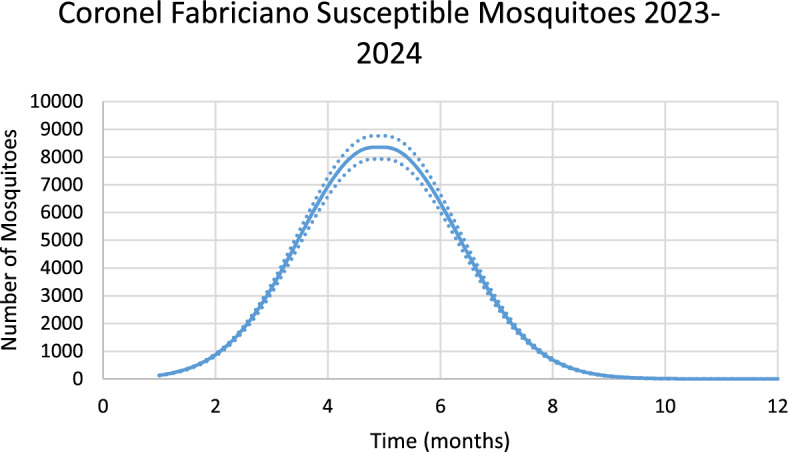
Estimated effective number of susceptible mosquitoes involved in the season’s outbreak in Coronel Fabriciano city. Continuous line represents the average number of mosquitoes and dotted lines represent the 95% Confidence Interval. The best fit GMF parameters were A = 15,311.6 (95% CI ± 765.6); µ = 4.96(95% CI ± 0.25); ${\sigma }_{L}$ = 0.05 (95% CI ± 0.003); ${\sigma }_{1}$ = 3.0 (95% CI ± 0.15); ${\sigma }_{2}$ = 0.87 (95% CI ± 0.04) (Color figure online)

Figure 6 shows the estimated effective number of susceptible mosquitoes involved in the season’s outbreak.

Note that there is a 2-month delay between the peak in the number of susceptible mosquitoes and the peak in the number of dengue cases.

**Fig. 7:**
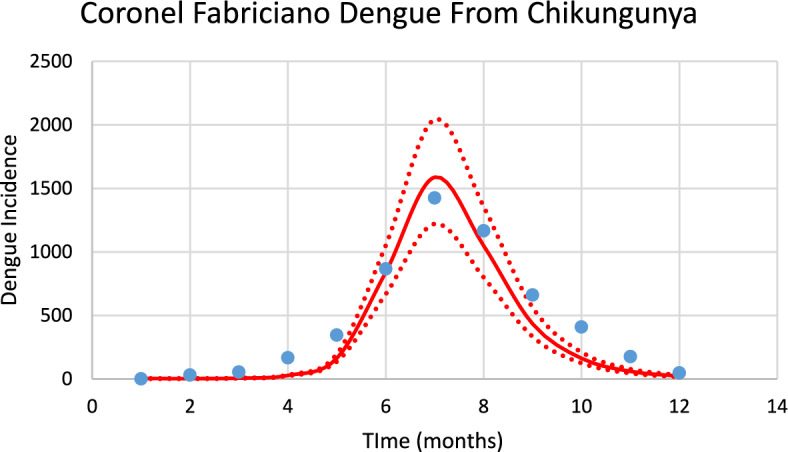
Model performance for dengue incidence in mosquitoes from chikungunya in Coronel Fabriciano city (R^2^ = 0.84). Dotted lines represent the Confidence Interval varying the size of the mosquitoes’ prevalence by 5% (Color figure online)

In Coronel Fabriciano, during the 2023–2024 transmission season, there were simultaneous outbreaks of dengue and chikungunya, although the number of cases in the latter was much lower than that in the former. Since the same mosquito transmits both infections and since we have the estimated number of mosquitoes, we checked the model by calculating the dengue incidence with the mosquitoes calculated from chikungunya. In order to calculate the dengue outbreak from the mosquitoes calculated from chikungunya, we modified the following parameters: ${\gamma }_{H}, c, b$ (see Table 1), and *n*_*m*_ calculated taking into account the history of dengue infection in this city. Figure 7 shows the results.

The number of observed dengue cases during the outbreak was 5378, whereas the total number of calculated cases was 5129.

Since, as mentioned above, the number of mosquitoes (prevalence) is rarely measured, it is convenient to carry out a sensitivity analysis of the number of human cases to each of the GMF parameters.

The sensitivity analysis consists in calculating the so-called “elasticity”, which gives the percentage change, for example, in a quantity $Q$ (below, number of human cases) with respect to the percentage change in each parameter ${p}_{i}$ (below, the parameters of the GMF). Therefore, the elasticity of the quantity $Q$ (number of human cases) with respect to each parameter ${p}_{i}$ is given by:

$${\varepsilon }_{Q}^{{p}_{i}}=\frac{\partial Q}{\partial {p}_{i}}\frac{{p}_{i}}{Q}\approx \frac{\%\Delta Q}{\%\Delta {p}_{i}}$$

Hence, varying each parameter of the GMF in 1% results in: 5% variation in humans cases for dengue and 4% for chikungunya, for parameter $A$; 0.5% for parameter $\mu$ for dengue and 0.7% for chikungunya; 0.1% for parameter ${\sigma }_{L}$ for dengue and 0.08% for chikungunya; 2,2% for parameter ${\sigma }_{1}$ for dengue and 1.7% for chikungunya; and 1.2% for parameter ${\sigma }_{2}$ for dengue and 1.9% for chikungunya.

### Pirapora City

**Fig. 8:**
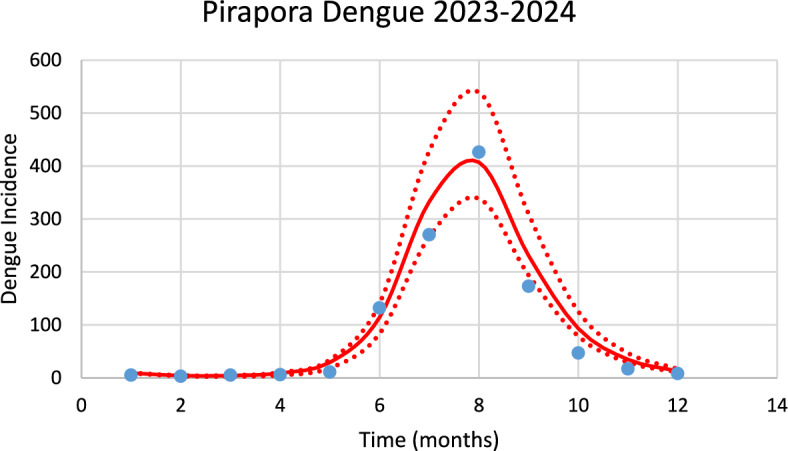
Model’s performance in reproducing the real data of dengue incidence for Pirapora city in the 2023–2024 transmission season (R^2^ = 0.92). Blue dots represent real data, red line the model’s result and dotted line the Confidence Interval varying the size of the mosquitoes’ prevalence by 5% [*Incidence* (*t*) in Eq. (2)] (Color figure online)

Figure 8 shows the model’s performance in fitting the real data of dengue incidence for Pirapora city in the 2023–2024 season of transmission.

The number of observed dengue cases during the outbreak was 1104, whereas the total number of calculated cases was 1178.

**Fig. 9:**
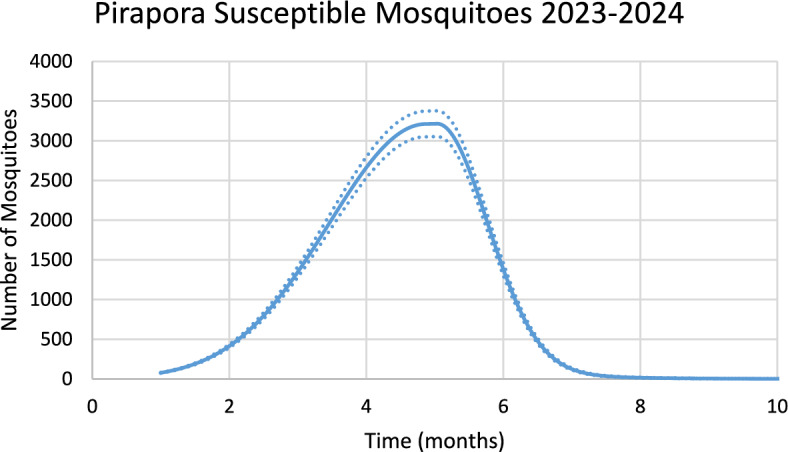
Estimated effective number of susceptible mosquitoes involved in the 2023–2024 season outbreak. Continuous line represents the average number of mosquitoes and dotted lines represent the 95% Confidence Interval. The best fit GMF parameters were A = 11,342.2 (95% CI ± 576.1); µ = 4.2 (95% CI ± 0.21); ${\sigma }_{L}$ = 0.09 (95% CI ± 0.005); ${\sigma }_{1}$ = 2.6 (95% CI ± 0.13); ${\sigma }_{2}$ = 0.4 (95% CI ± 0.02) (Color figure online)

Figure 9 shows the estimated effective number of mosquitoes (prevalence) involved in the season’s outbreak.

Again, there is a 2-month delay between the peak in the number of susceptible mosquitoes and the peak in the number of dengue cases.

**Fig. 10:**
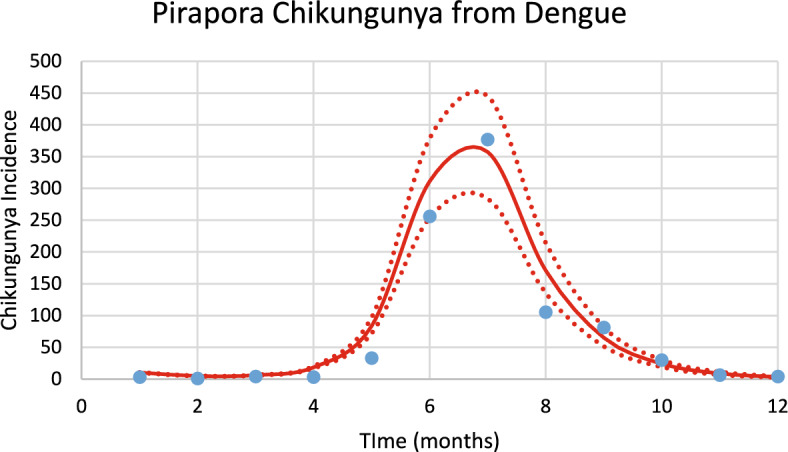
Model’s performance for incidence of chikungunya in Pirapora city using the number of mosquitoes calculated from dengue incidence for the 2023–2024 transmission season (R^2^ = 0.94). Dotted lines represent the Confidence Interval varying the size of the mosquitoes’ prevalence by 5% (Color figure online)

Figure 10 shows the incidence of chikungunya in Pirapora city using the number of mosquitoes calculated from dengue incidence for the 2023–2024 transmission season and modifying the parameters ${\gamma }_{H}, c, b$ (see Table 1), and *n*_*m*_ calculated taking into account the history of chikungunya infection in this city.

The number of observed chikungunya cases during the outbreak was 353, whereas the total number of calculated cases was 396.

For Pirapora, varying each parameter of the GMF in 1% results in: 4% variation in humans cases for dengue and 4% for chikungunya, for parameter $A$; 3.4% for parameter $\mu$ for dengue and 3.5% for chikungunya; 0.22% for parameter ${\sigma }_{L}$ for dengue and 0.19% for chikungunya; 2,5% for parameter ${\sigma }_{1}$ for dengue and 2.2% for chikungunya; and 0.7% for parameter ${\sigma }_{2}$ for dengue and 0.6% for chikungunya.

### Teófilo Otoni City

The reader surely noted that to calculate the mosquitoes’ population during the outbreak, we can choose either dengue or chikungunya data. Typically, we choose the largest outbreak and then we calculate the mosquitoes’ population from the dengue outbreak.

**Fig. 11:**
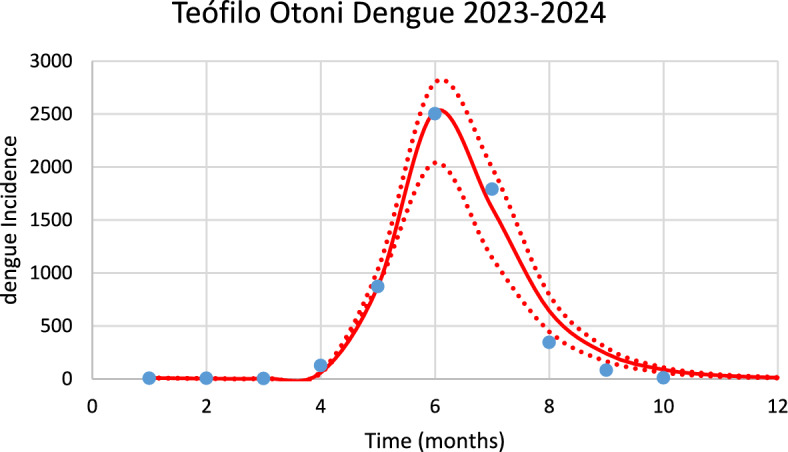
Model’s performance in fitting the real data of chikungunya incidence for Teófilo Otoni city in the 2023–2024 season of transmission (R^2^ = 0.99). Blue dots represent real data, red line the model’s result and dotted lines the Confidence Interval varying the size of the mosquitoes’ prevalence by 5% [*Incidence* (*t*) in Eq. (1)] (Color figure online)

Figure 11 shows the model’s performance in reproducing the real data of dengue incidence for Teófilo Otoni city in the 2023–2024 season of transmission.

The number of observed dengue cases during the outbreak was 5740, whereas the total number of calculated cases was 6098.

**Fig. 12:**
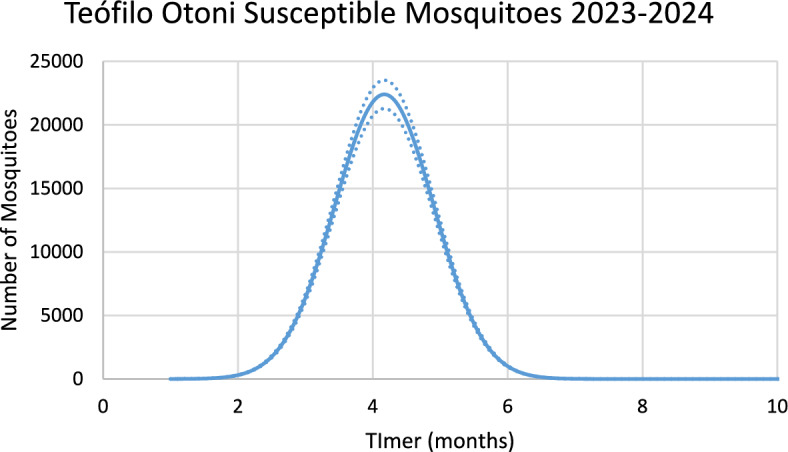
Estimated effective number of susceptible mosquitoes involved in the season’s outbreak for Teófilo Otoni city with 95% Confidence Interval. The best fit GMF parameters were A = 3200 (95% CI ± 160); µ = 4.8 (95% CI ± 0.24); ${\sigma }_{L}$ = 0.18 (95% CI ± 0.009); ${\sigma }_{1}$ = 2.0 (95% CI ± 0.1); ${\sigma }_{2}$ = 0.5 (95% CI ± 0.025) (Color figure online)

Figure 12 shows the estimated effective number of susceptible mosquitoes involved in the season’s outbreak.

Again, there is a 2-month delay between the peak in the number of susceptible mosquitoes and the peak in the number of chikungunya cases.

**Fig. 13:**
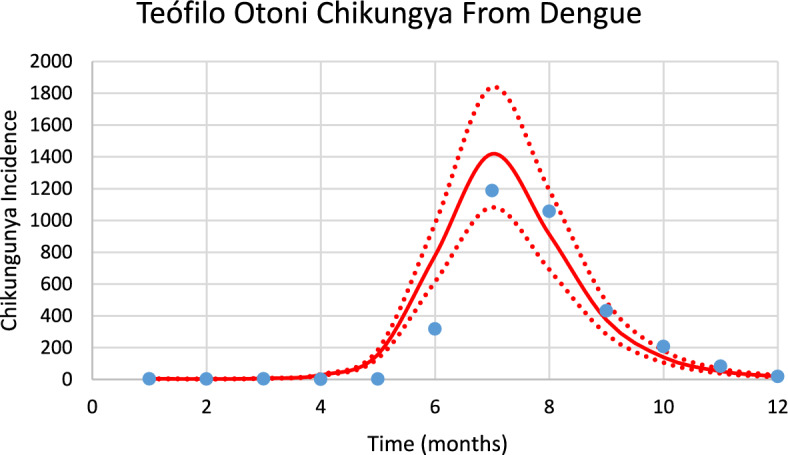
Model’s performance for incidence of chikungunya in Teófilo Otoni city using the number of mosquitoes calculated from dengue incidence for the 2023–2024 transmission season (R^2^ = 0.92). Dotted lines represent the Confidence Interval varying the size of the mosquitoes’ prevalence by 5% (Color figure online)

Figure 13 shows the incidence of chikungunya in Teófilo Otoni using the number of mosquitoes calculated from dengue incidence for the 2023–2024 transmission season and modifying the parameters ${\gamma }_{H}, c, b$ (see Table 1), and *n*_*m*_ calculated taking into account the history of chikungunya infection in this city.

The number of observed chikungunya cases during the outbreak was 718, whereas the total number of calculated cases was 735.

For Teófilo Otoni, varying each parameter of the GMF in 1% results in: 4.4% variation in humans cases for dengue and 4% for chikungunya, for parameter $A$; 1% for parameter $\mu$ for dengue and 0.3% for chikungunya; 0.18% for parameter ${\sigma }_{L}$ for dengue and 0.12% for chikungunya; 1.65% for parameter ${\sigma }_{1}$ for dengue and 1.5% for chikungunya; and 0.86% for parameter ${\sigma }_{2}$ for dengue and 0.62% for chikungunya.

### Paracatú City

To demonstrate that the order of the infections is not important, for this city we also calculated the mosquitoes’ population from the chikungunya outbreak rather than that from the dengue outbreak.

**Fig. 14:**
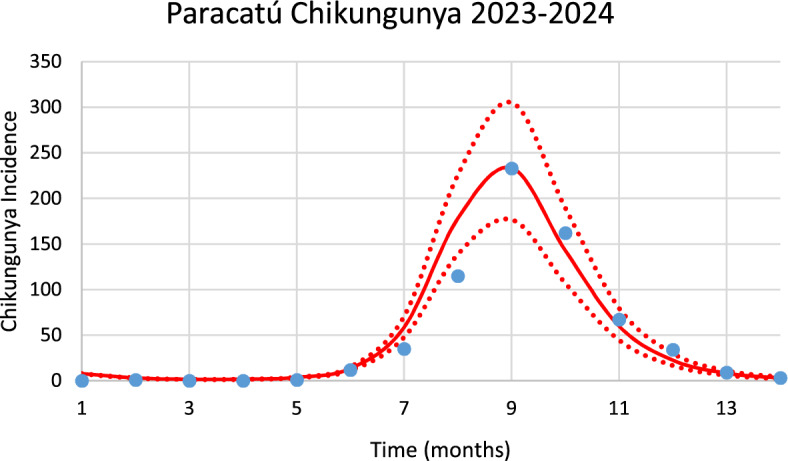
Model performance in reproducing the real data of chikungunya incidence for the city of Paracatú in the 2023–2024 season of transmission (R^2^ = 0.93). Blue dots represent real data, red line the model’s result and dotted lines the Confidence Interval varying the size of the mosquitoes’ prevalence by 5% [*Incidence* (*t*) in Eq. (1)] (Color figure online)

Figure 14 shows the model’s performance in reproducing the real data of chikungunya incidence for the city of Paracatú in the 2023–2024 season of transmission.

The number of observed chikungunya cases during the outbreak was 672, whereas the total number of calculated cases was 732.

**Fig. 15:**
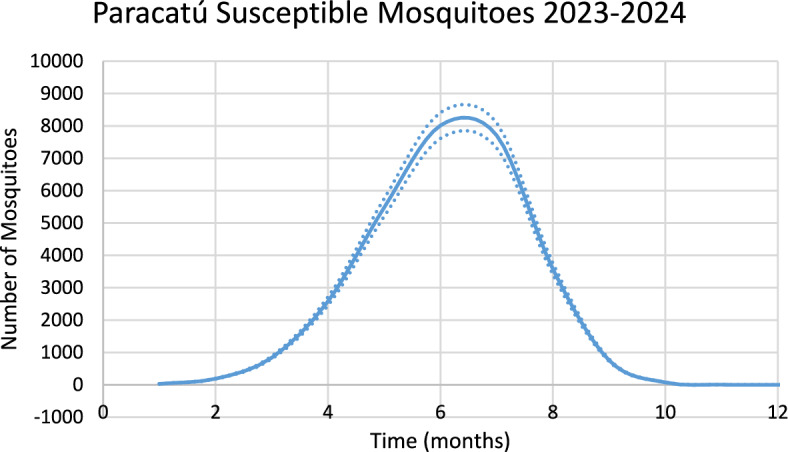
Estimated effective number of susceptible mosquitoes involved in the season outbreak in the city of Paracatú. Dotted lines represent the 95% Confidence Interval. The best fit GMF parameters were A = 8126.6 (95% CI ± 406.3); µ = 6.3 (95% CI ± 0.32); ${\sigma }_{L}$ = 0.005 (95% CI ± 0.0003); ${\sigma }_{1}$ = 2.7 (95% CI ± 0.14); ${\sigma }_{2}$ = 1.3 (95% CI ± 0.07) (Color figure online)

Figure 15 shows the estimated effective number of susceptible mosquitoes involved in the season’s outbreak.

Again, there is a 2-month delay between the peak in the number of susceptible mosquitoes and the peak in the number of chikungunya cases.

**Fig. 16:**
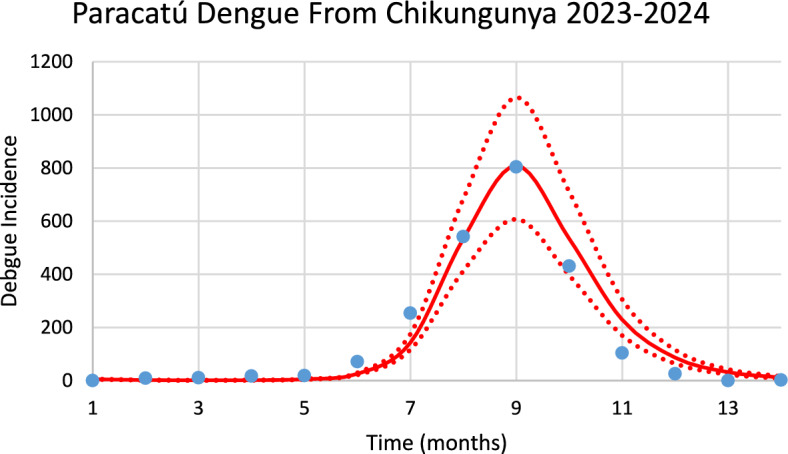
Model’s performance for incidence of dengue in Paracatú city using the number of mosquitoes calculated from the chikungunya incidence for the 2023–2024 transmission season (R^2^ = 0.97). Dotted lines represent the Confidence Interval varying the size of the mosquitoes’ prevalence by 5% (Color figure online)

Figure 16 shows the incidence of dengue in Paracatú using the number of mosquitoes calculated from chikungunya incidence for the 2023–2024 transmission season and modifying the parameters ${\gamma }_{H}, c, b$ (see Table 1), and *n*_*m*_ calculated taking into account the history of dengue infection in this city.

The number of observed dengue cases during the outbreak was 2266, whereas the total number of calculated cases was 2310.

For Paracatú, varying each parameter of the GMF in 1% results in: 5.6% variation in humans cases for dengue and 5.3% for chikungunya, for parameter $A$; 4.5% for parameter $\mu$ for dengue and 5.4% for chikungunya; 0% for parameter ${\sigma }_{L}$ for dengue and 0% for chikungunya; 3.2% for parameter ${\sigma }_{1}$ for dengue and 3% for chikungunya; and 1.4% for parameter ${\sigma }_{2}$ for dengue and 1.1% for chikungunya.

### Aimorés City

In this city, for the season analyzed, the number of chikungunya cases (380) was greater than that of dengue cases (276). This city is small (25,269 inhabitants), and the cumulative number of reported dengue cases in its whole history totals 3751, almost 15% of the population, whereas the cumulative number of reported chikungunya cases is only 915. As explained in the Methods section, this fact approximately mimics herd immunity and was taken into account in the calculations. This explains why the number of chikungunya cases for the season analyzed was greater than that of dengue cases, at least in part.

**Fig. 17:**
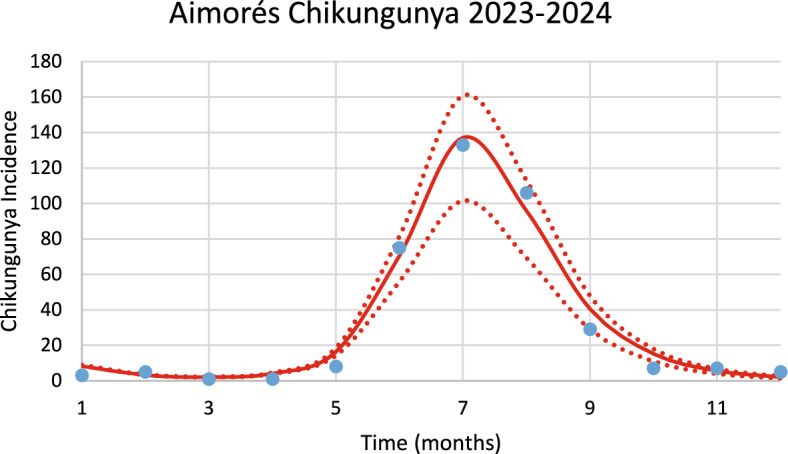
Model’s performance in reproducing the real data of chikungunya incidence for the city of Aimorés in the 2023–2024 season of transmission (R^2^ = 0.98). Blue dots represent real data, red line the model’s result and dotted lines the Confidence Interval varying the size of the mosquitoes’ prevalence by 5% [*Incidence* (*t*) in Eq. (1)] (Color figure online)

Figure 17 shows the model’s performance in reproducing the real data of chikungunya incidence for the city of Aimorés in the 2023–2024 season of transmission.

The number of observed chikungunya cases during the outbreak was 380, whereas the total number of calculated cases was 395.

**Fig. 18:**
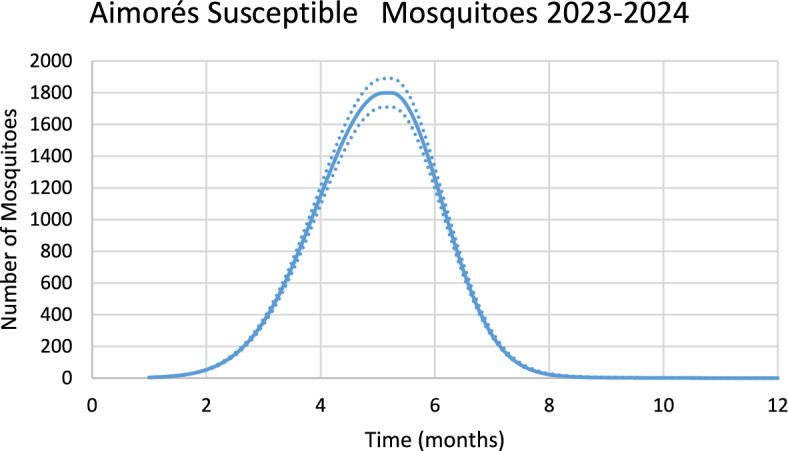
Estimated effective number of susceptible mosquitoes involved in the season’s outbreak for the city of Aimorés. Dotted lines represent the 95% Confidence Interval. The best fit GMF parameters were A = 1796.2 (95% CI ± 89.6); µ = 5.0 (95% CI ± 0.25); ${\sigma }_{L}$ = 0.2 (95% CI ± 0.01); ${\sigma }_{1}$ = 1.4 (95% CI ± 0.07); ${\sigma }_{2}$ = 0.8 (95% CI ± 0.04) (Color figure online)

Figure 18 shows the estimated effective number of susceptible mosquitoes involved in the season’s outbreak.

Again, there is a 2-month delay between the peak in the number of susceptible mosquitoes and the peak in the number of chikungunya cases.

**Fig. 19:**
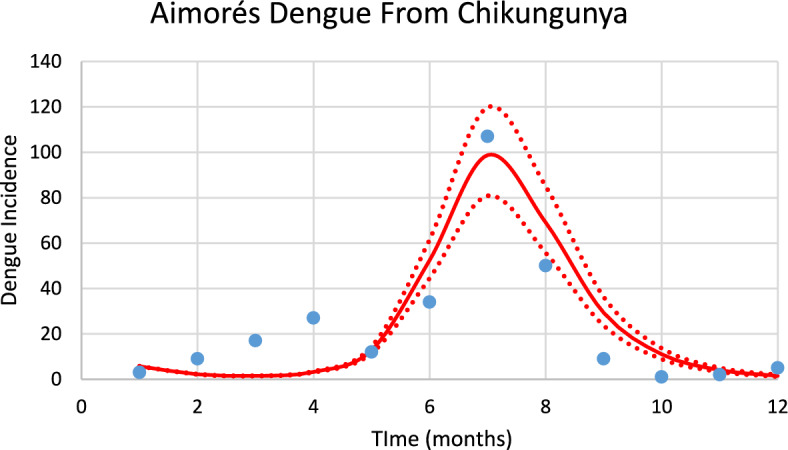
Model’s performance for incidence of dengue in Aimorés city using the number of mosquitoes calculated from the chikungunya incidence for the 2023–2024 transmission season (R^2^ = 0.82). Dotted lines represent the Confidence Interval varying the size of the mosquitoes’ prevalence by 5% (Color figure online)

Figure 19 shows the incidence of dengue in Aimorés using the number of mosquitoes calculated from chikungunya incidence for the 2023–2024 transmission season and modifying the parameters ${\gamma }_{H}, c, b$ (see Table 1), and *n*_*m*_ calculated taking into account the history of dengue infection in this city.

The number of observed dengue cases during the outbreak was 276, whereas the total number of calculated cases was 287.

For Aimorés, varying each parameter of the GMF in 1% results in: 3.8% variation in humans cases for dengue and 4% for chikungunya, for parameter $A$; 3.5% for parameter $\mu$ for dengue and 3.9% for chikungunya; 0.15% for parameter ${\sigma }_{L}$ for dengue and 0.15% for chikungunya; 2% for parameter ${\sigma }_{1}$ for dengue and 2% for chikungunya; and 0.9% for parameter ${\sigma }_{2}$ for dengue and 0.9% for chikungunya.

Before proceeding for the next analysis, it is worth noting that the uniformly narrow 95% CIs for GMF parameters across all cases studied support practical identifiability in this data context.

The number of susceptible mosquitoes infected with both dengue and chikungunya viruses in each city and their correlation with the observed incidence rates.

The model calculates the number of infected mosquitoes in each outbreak. This number should be linearly correlated with the number of cases of both dengue and chikungunya infections. Below, we present figures showing these correlations for both infections and then analyze the slopes of the correlation lines, which give the number of infected persons per unit of variation in the number of infected mosquitoes.

Figures 20, 21, 22, 23, 24, 25, 26, 27, 28, and 29 show the results of the correlations.

**Fig. 20:**
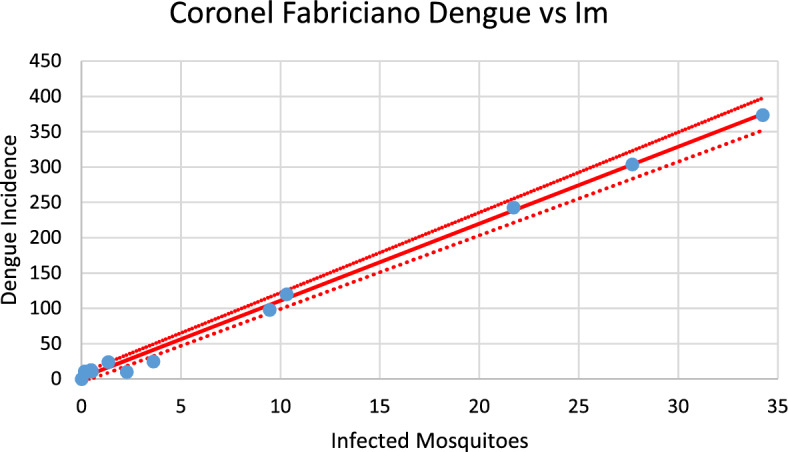
Correlation between infected mosquitoes and the incidence of dengue for Coronel Fabriciano (R^2^ = 0.995). Blue dots represent real data continuous line the model’s result and dotted lines the 95% Confidence Interval. The fitting parameter is: Slope = 10.903 (10.431–11,374) (Color figure online)

**Fig. 21:**
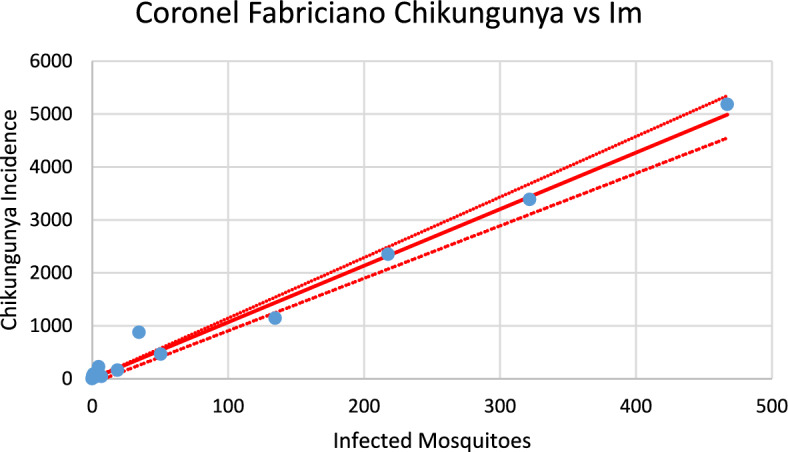
Correlation between infected mosquitoes and the incidence of chikungunya for Coronel Fabriciano (R^2^ = 0.986). Blue dots represent real data continuous line the model’s result and dotted lines the 95% Confidence Interval. The fitting parameter is: Slope = 10.608 (9.918–11,443) (Color figure online)

**Fig. 22:**
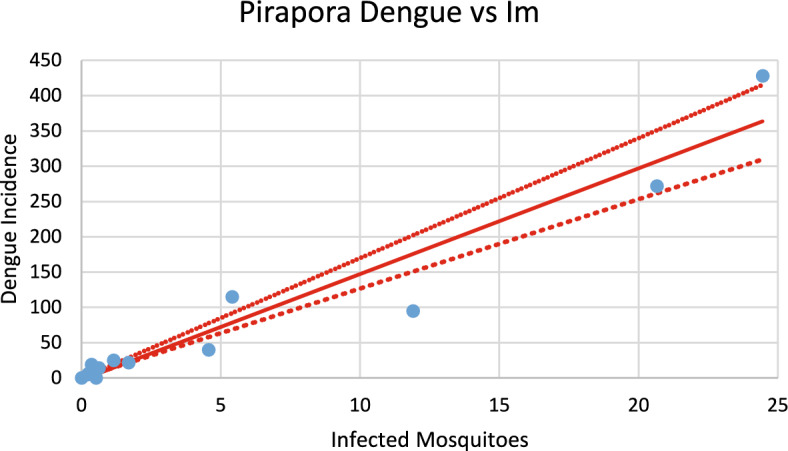
Correlation between infected mosquitoes and the incidence of dengue for Pirapora (R^2^ = 0.921). Blue dots represent real data continuous line the model’s result and dotted lines the 95% Confidence Interval. The fitting parameter is: Slope = 14.824 (12.663–16.986) (Color figure online)

**Fig. 23:**
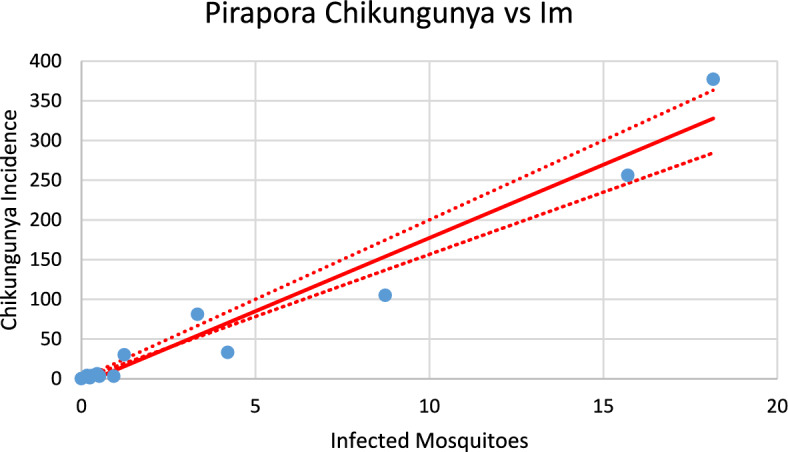
Correlation between infected mosquitoes and the incidence of chikungunya for Pirapora (R^2^ = 0.951). Blue dots represent real data continuous line the model’s result and dotted lines the 95% Confidence Interval. The fitting parameter is: Slope = 17.891 (15.664–20.097) (Color figure online)

**Fig. 24:**
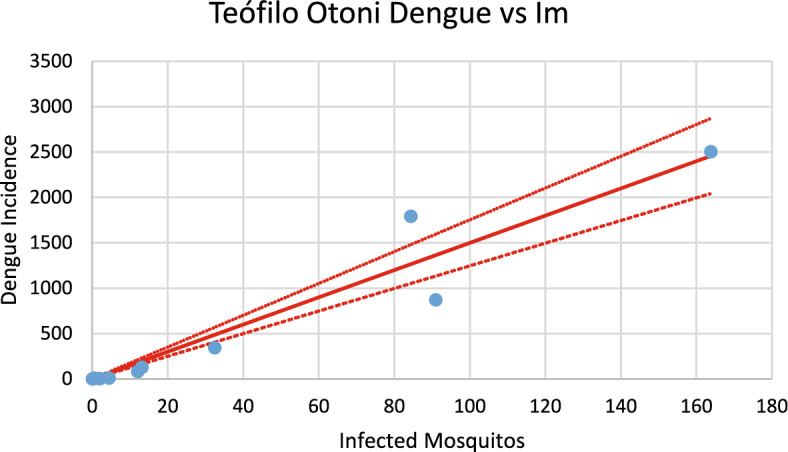
Correlation between infected mosquitoes and the incidence of dengue for Teófilo Otoni (R^2^ = 0.926). Blue dots represent real data, continuous line the model’s result and dotted lines the 95% Confidence Interval. The fitting parameter is: Slope = 14.997 (12,471–17.523) (Color figure online)

**Fig. 25:**
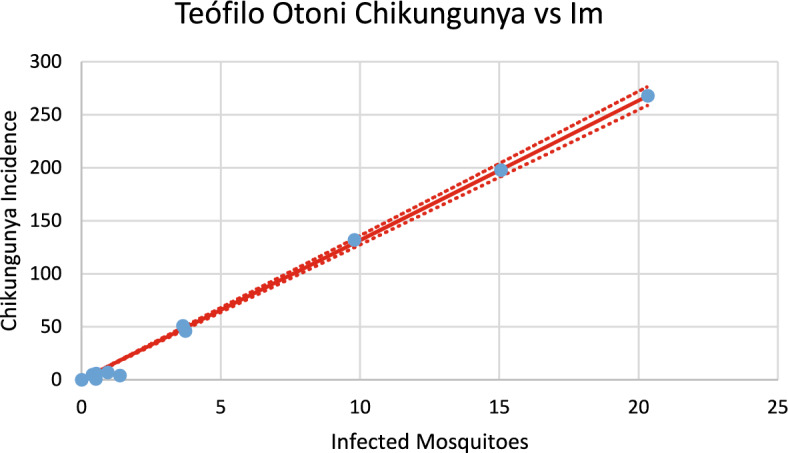
Correlation between infected mosquitoes and the incidence of chikungunya for Teófilo Otoni (R^2^ = 0.998). Blue dots represent real data, continuous line the model’s result and dotted lines the 95% Confidence Interval. The fitting parameter is: Slope = 13.172 (12,738–13.606) (Color figure online)

**Fig. 26:**
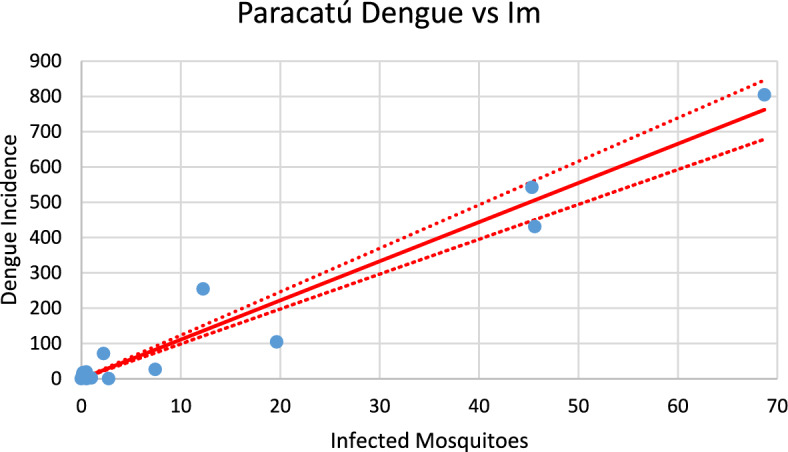
Correlation between infected mosquitoes and the incidence of dengue for Paracatú (R^2^ = 0.95). Blue dots represent real data, continuous line the model’s result and dotted lines the 95% Confidence Interval. The fitting parameter is: Slope = 11.099 (9.576–12.622) (Color figure online)

**Fig. 27:**
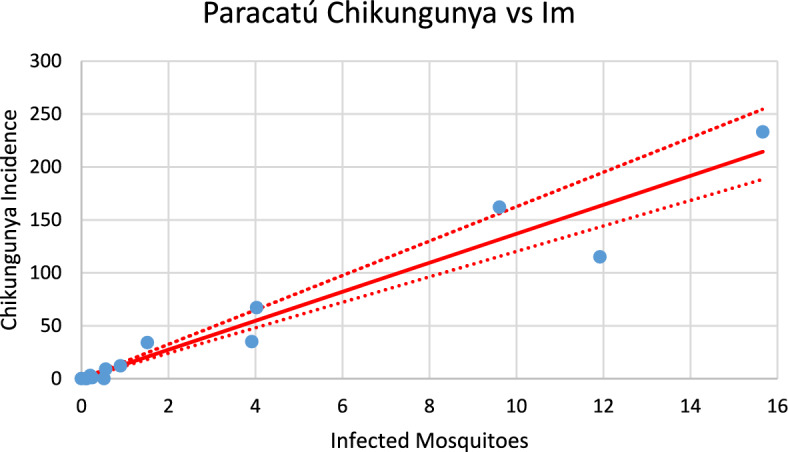
Correlation between infected mosquitoes and the incidence of chikungunya for Paracatú (R^2^ = 0.951). Blue dots represent real data, continuous line the model’s result and dotted lines the 95% Confidence Interval. The fitting parameter is: Slope = 13,521 (10,789–16,252) (Color figure online)

**Fig. 28:**
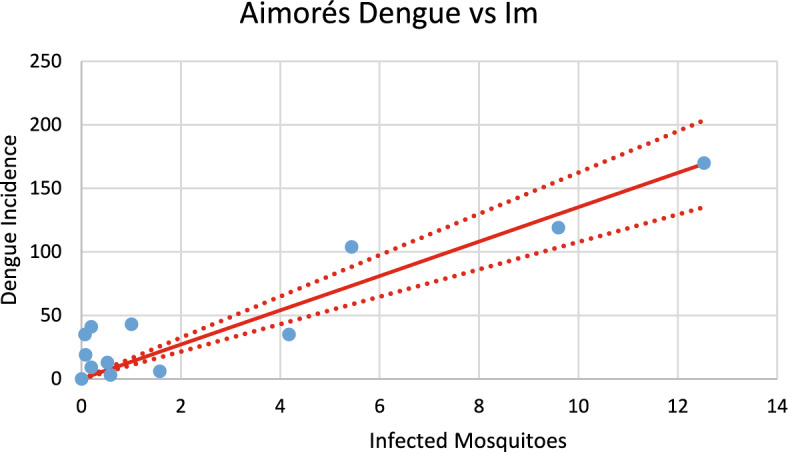
Correlation between infected mosquitoes and the incidence of dengue for Aimorés (R^2^ = 0.91). Blue dots represent real data, continuous line the model’s result and dotted lines the 95% Confidence Interval. The fitting parameter is: Slope = 13.521 (10.789–16.252) (Color figure online)

**Fig. 29:**
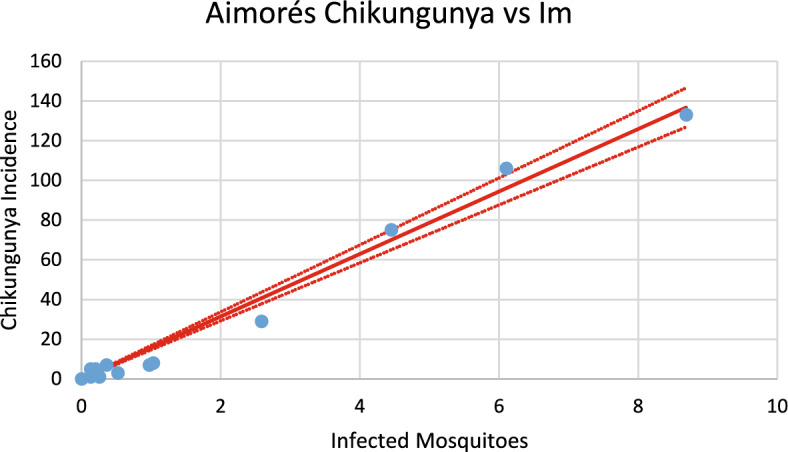
Correlation between infected mosquitoes and the incidence of chikungunya for Aimorés (R^2^ = 0.987). Blue dots represent real data, continuous line the model’s result and dotted lines the 95% Confidence Interval. The fitting parameter is: Slope = 15.739 (14.601–16.873) (Color figure online)

From the calculations performed to estimate the number of cases against the number of infected mosquitoes, we can ask how many cases of each infection should be expected from one infected mosquito. This follows from the slope of the regression lines [Eq. (13)]. By looking at the figures, one should expect great differences but, as shown below, the slopes differences are not significant. Table 2 summarizes the results of the above analysis.

**Table 2:** Slopes of the correlation between Infected mosquitoes and incidences

	Chikungunya	Dengue
	Slopes	
Coronel fabriciano	10.680 (9.918–11.443)	10.903 (10.431–11.374)
Pirapora	17.891 (15.664–20.097)	14.824 (12.663–16.986)
Teófilo Otoni	13.172 (12.738–13.606)	14.997 (14.471–17.523)
Paracatu	13.687 (12.027–19.347)	11.099 (9.570–12.622)
Aimorés	15.739 (14.605–16.873)	13.521 (10.789–16.252)

Note that the confidence intervals overlap, meaning that the differences between the dengue and chikungunya slopes are not statistically significant (*p* = 0.58). This can be understood if, on the one hand, dengue is more infective than chikungunya is (${b}_{dengue}>{b}_{chik}$), but on the other hand, the fraction of individuals susceptible to dengue is lower than that for chikungunya $\left(\frac{{S}_{{H}_{dengue}}}{{N}_{H}}<\frac{{S}_{{H}_{chik}}}{{N}_{H}}\right)$, and one effect compensates for the other effect.

It is also possible from the above calculation to calculate the probability of having a mosquito infected by two infections simultaneously, assuming that this biologically feasible by the principle of competitive exclusion [see Amaku et al. (2010a, b), Burattini et al. (2008)]. Anyway, this probability is very low, of the order of 0.002%.

### Checking the Model Against Empirical Data of Mosquitoes in Foz do Iguaçu, Paraná, Brazil

In 2024, the City of Foz do Iguaçu published a document with the statistics of, among other things, data of Aedes mosquitoes collected in the city in 2017 (Rodrigues et al. 2017). We used dengue incidence data for that year to calculate the number of susceptible mosquitoes in the same region where mosquitoes counting took place. The total number of mosquitoes counted in the area was 52,401, whereas the total number calculated with Model (2–12) was 58,694, which shows that the model tallies empirical data reasonably well, although, with 285,415 inhabitants, Foz do Iguaçú is a comparatively large city.

**Fig. 30:**
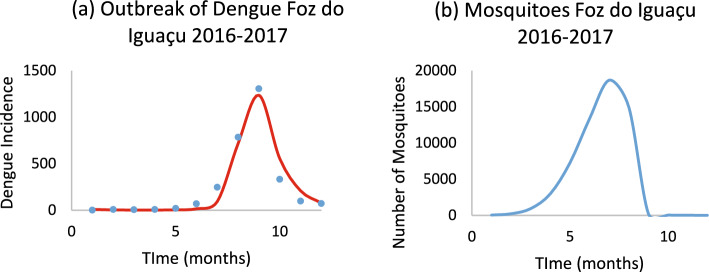
Model fitting to dengue data (a) and the calculated number of Aedes mosquitoes (b) for the city of Foz do Iguaçu. In **a** blue dots represent real data and red line the model’s result [*Incidence* (*t*) in Eq. (1)] (Color figure online)

Figure 30 shows the model’s fit to dengue data (a) and the calculated number of Aedes mosquitoes (b) for the city of Foz do Iguaçu.

## Discussion

In this work, we propose a theoretical method to estimate the number of Aedes mosquitoes involved in outbreaks of dengue and chikungunya by assuming that the population of susceptible mosquitoes varies in time according to a Gaussian Mesa Function due to climatic factors. We calculated the parameters of this distribution to reproduce it using a modified Ross‒Macdonald model [see Eq. (2)] and the incidence curves of both infections studied. We use a flexible GMF to model mosquitoes’ population dynamics during an outbreak that captures the complex incidence patterns similarly to the sub-epidemic framework. While the GMF approach uses a single flexible curve, it can approximate the complex multi-wave epidemic shapes observe in real-world data. Of course, other functions could be tried, but we found, after trying a few, that the GMF is the one that produces the best fit of the calculated incidence to the observed incidence data and that reproduces the observed shape of mosquitoes (see Fig. 30).

Our findings have important biological interpretations. The magnitude of the mosquitoes’ population outbreaks determines the size of the disease outbreaks. Therefore, the size of the mosquitoes’ outbreak is a crucial variable related to control, strongly supporting the findings of Amaku et al. (2014), who calculated the sensitivity of dengue outbreak with simple Ross‒Macdonald model, to each of the parameters related to transmission. The authors (Amaku et al. 2014) concluded the most effective measure to control dengue outbreaks is to reduce the size of adult mosquitoes’ population.

It is well known from the literature that rain and temperature are the main climatic factors influencing mosquitoes’ population size (http://www.ipardes.gov.br/cadernos/MontaCadPdf1.php?Municipio=85850^2024^). A previous theoretical paper (Coutinho et al. 2006) investigated the relationships among climatic factors, mosquitoes’ populations and dengue outbreaks. In the present paper, however, no climate model was proposed, and we used only empirical data on the incidence of outbreaks of dengue and chikungunya for several cities in Brazil, exemplifying the model with five municipalities from the State of Minas Gerais, a state with a high incidence of dengue and chikungunya. In a future paper, we will relate the findings of this current paper with climatic data.

We observed a delay between the peak in the mosquitoes’ population and both the peak in the infected mosquitoes’ population and the peak in the incidence of the infections studied.

In addition, we found a linear correlation between the number of infected mosquitoes’ and the incidence of infections. As mentioned above, the slope of that linear correlation is related to the incidence according to Eq. (3). Although dengue and chikungunya differ with respect to their degree of infection, the slopes of the correlation between infected mosquitoes and incidence did not differ statistically, both among the cities and between the infections. This can be understood, as mentioned above, if we consider that, on the one hand, dengue is more infective than chikungunya is (${b}_{dengue}>{b}_{chik}$), but on the other hand, the fraction of individuals susceptible to dengue is lower than that for chikungunya $\left(\frac{{S}_{{H}_{dengue}}}{{N}_{H}}<\frac{{S}_{{H}_{chik}}}{{N}_{H}}\right)$, and one effect compensates for the other. Another factor that could interfere with these results would be the size of the cities, but the selected sites chosen to illustrate the model were similar in population size and in the same geographical region of the country, with a similar climate.

Some limitations of the model are worth mentioning. First, the model assumes that the human population is homogeneously distributed and that the contact with the vectors is with the entire population of each city. These assumptions may be justified because humans are very mobile and travel along the entire geographical limits of cities. Another limitation is that the model is deterministic and ignores stochasticity. In addition, the values of the parameters related to the infection, although taken from the literature, are subject to uncertainties that were neglected in this paper.

In conclusion, our model shows that it is possible to estimate the size of the mosquitoes’ population from incidence data, circumventing the logistic hurdles involved in the actual counting of mosquitoes.
